# A Novel Type-I Interferon Family, Bovine Interferon-Chi, Is Involved in Positive-Feedback Regulation of Interferon Production

**DOI:** 10.3389/fimmu.2020.528854

**Published:** 2020-10-30

**Authors:** Yongli Guo, Zhifeng Song, Chenfeng Li, Yueyang Yu, Haiyue Dai, Xiuxin Luo, Yujiao Wang, Junwei Wang, Mingchun Gao

**Affiliations:** ^1^ College of Veterinary Medicine, Northeast Agricultural University, Harbin, China; ^2^ Animal Disease Prevention and Control Center of Heilongjiang Province, Harbin, China

**Keywords:** bovine IFN-χ, molecular characterization, antiviral activity, JAK-STAT signaling pathway, positive feedback regulation

## Abstract

Interferon-chi (IFN-χ) is a type of function-unknown IFN. IFN-χ in bovines (BoIFN-χ) has evolved as a multigene family. This family comprises four IFN-χ subtypes, two of which are functional genes, which we demonstrated to (i) have antiviral and antiproliferative activities, (ii) be highly sensitive to trypsin, and (iii) remain stable with changes in pH and temperature. BoIFN-χ is a key intermediate in antiviral response, PAbs against BoIFN-χs could downregulate the transcriptional activation of ISGs induced by poly(I:C), and BoIFN-χs could be induced upon virus infection at the early and late phase. Additionally, BoIFN-χs bind with type-I IFN receptors, induce transcription of interferon regulatory factor 7 (IRF7), interferon-stimulated genes (ISGs), and type-I IFNs as well as myxovirus resistance protein 1 (Mx1) expression. Expression of ISGs and activation of IFN-stimulated response element (ISRE) induced with BoIFN-χs could be downregulated significantly by the Janus kinase (JAK) 1 and signal transducers and activators of transcription (STAT) 1 inhibitor. The promoters of BoIFN-β, nuclear factor-kappa B, and ISRE could be activated with BoIFN-χs, and the BoIFN-χ promoter could be activated by other type-I IFNs. Overall, BoIFN-χ could be induced with virus infection and signal through the JAK-STAT pathway to form a positive-feedback regulation of IFN production. These findings may facilitate further research on the role of IFN-χ in innate immune responses.

## Introduction

Interferons (IFNs) are a group of signaling proteins produced and secreted by host cells in response to the presence of pathogens (e.g., viruses, bacteria, parasites) and tumor cells. They have antiviral, antiproliferative, and immunomodulatory properties (1). IFNs are classified into types I, II, and III according to sequence homology and cell receptors.

Type-I IFN is the most studied and includes α, β, ω, κ, ϵ, and τ subclasses (2). It transduces intracellular signals by binding to the receptors IFNAR1 and IFNAR2 on the cell surface (3, 4), signals through the Janus kinase/signal transducers and activators of transcription (JAK-STAT) pathway, and induces production of IFN-stimulated genes (ISGs) to exert antiviral activity.

IFN-χ [also termed IFN-αω or IFN-μ (2, 5, 6)] is a new subclass of type-I IFN. It is quite distinct from IFN-α and IFN-ω (6, 7). IFN-χ has not been found in primates or rodents (despite searching the extensive genomic data available for several species) or in marsupials or monotremes (7). However, full-length open-reading frames (ORFs) of IFN-χ sequences have been identified in the genomes of cattle, sheep, and pigs as well as in the partial genomes of dogs, cats, hedgehogs, bats, flying foxes, and alpacas, suggesting that IFN-χ is widespread in Laurasiatheria (6). IFN-χ in pigs has only a single copy, which shows 65% nucleotide identity and 50% amino acid similarity to the closest subclass and exerts effective antiviral activity against the foot and mouth disease virus (FMDV) pre- and postinfection (7, 8).

Bovine IFN-χ (BoIFN-χ) research has been restricted to database analyses, which has been analyzed based on incomplete data on the bovine genome (5). Here, we characterize and measure the activity and regulation of the BoIFN-χ family. We find that BoIFN-χs have the typical molecular characteristics of type-I IFNs; exhibit antiviral and antiproliferative activities; signal through binding with the type-I IFN receptor complex; induce ISGs, IFN regulatory factor 7 (IRF7), and type-I IFNs; and activate the promoters of BoIFN-β, nuclear factor-kappa B (NF-κB), and IFN-stimulated response element (ISRE).

## Materials and Methods

### Cells and Viruses

Madin–Darby bovine kidney (MDBK) cells, porcine kidney (PK)-15 cells, Madin–Darby canine kidney (MDCK) cells, and baby hamster kidney (BHK)-21 cells were preserved by our research team. Primary bovine testicular (BT) cells were prepared and preserved by our research team. Primary embryo bovine kidney (EBK) cells and primary embryo bovine lung (BL) cells were separately gifted from Dr. Li Yu and Fei Xue (Harbin Veterinary Research Institute, Chinese Academy of Agricultural Sciences, Harbin, China). The vesicular stomatitis virus (VSV) and bovine herpesvirus (BHV) were purchased from the China Institute of Veterinary Drug Control (Beijing, China).

### Plasmids

BoIFN-χ expression plasmids with a polyhistidine (His) tag or no tag on the N terminus were constructed separately with specific primers (**Table S1**). In the reporter luciferase assay, the IFN-χ reporter plasmid was constructed. IFN-β, NF-κB, and ISRE reporter plasmids, a pRL-TK control plasmid, and pRL-SV40-Renilla interference plasmids were constructed as described previously (9). All the constructs mentioned above were confirmed using DNA sequencing.

### Construction of Gene Loci

Based on a database showing the whole genome sequence of bovines (GenBank: NC_007306), the type-I IFN gene fragments that aligned with >90% identity to the IFN gene sequences published previously or similar IFN gene sequences of other animals were matched and marked using the BLAST algorithm (10). Two potential functional IFN-χ genes in bovines (BoIFN-χ1 and BoIFN-χ3) were located in the bovine chromosome 8. Then, BoIFN-χ genes were cloned and molecular characterizations undertaken using bioinformatics software, including ORF Finder algorithm, SignalP Server online, NetGlycate 1.0 Server online, Swiss-Model Server online, Lasergene 11 package, CLUSTALX, and MEGA 7.0.

### Protein Expression and Biologic Characteristics

Expression plasmids were transformed into *Escherichia coli Rosetta (DE3)*, the recombinant proteins were expressed with induction of isopropyl ß-D-1-thiogalactopyranoside. Proteins without tags were used as immune antigens to prepare polyclonal antibodies (PAbs) against BoIFN-χs. Proteins with a His tag were purified with Ni^+^-chelated columns for assessment of biologic characteristics.

The antiviral activities of BoIFN-χs were determined by inhibiting the cytopathic effect (CPE) on cells caused by a virus as described previously (11). The specific antiviral activities were measured with PAbs against BoIFN-χs. Antiproliferative activities were measured with the 3-(4,5-dimethylthiazol-2-yl)-2,5-diphenyltetrazolium bromide (MTT) assay as described previously (12). Physicochemical characteristics were assessed with a MDBK/VSV measurement system, which included sensitivity to trypsin, pH, and temperature (13).

### PAbs Preparation and the Specificity Assay

Recombinant protein of BoIFN-χ3 without tags was expressed and purified by gel cutting as described previously (14). Then, the purified protein was used as immune antigens to prepare PAbs against BoIFN-χs. The specificity and titer of PAbs were measured by indirect enzyme-linked immunosorbent assay (ELISA) with the recombinant protein with His-tag as coating protein and the preimmune serum was used as the control.

The inactive PAbs and BoIFN-χs were incubated at 37°C for 2 h and then the antiviral activity was assessed with a MDBK/VSV measurement system after centrifugation by CPE and TCID50 methods. The cells with preimmune serum or without PAbs were used as control.

BL cells were pretreated with the inactive PAbs for 2 h, and polyinosinic:polycytidylic acid (poly(I:C); 1 μg/mL) were then transfected for another 3, 6, or 9 h and the ISG transcription was detected by quantitative real-time polymerase chain reaction (qPCR).

### Receptor-Blocking Assay

ELISA plates were coated with recombinant receptor proteins (BoIFNAR1-EC and BoIFNAR2-EC, expressed and purified in the laboratory previously) (11) overnight at 4°C. Then, the ELISA plates were blocked with 5% skimmed milk/phosphate-buffered saline with Tween 20 (PBST) for 2 h at room temperature. Serial dilutions of recombinant proteins were added before addition of PAbs against BoIFN-χs. Horseradish peroxidase (HRP)-conjugated goat antirabbit immunoglobulin (Ig)G was used as the secondary antibody. Reactivity was visualized by color development using the chromogen/substrate mixture 3,3′,5,5′-Tetramethylbenzidine/H_2_O_2_ (Sigma–Aldrich, Saint Louis, MO, USA). After 10–15 min, the reaction was stopped with the addition of 1.0 M H_2_SO_4_. The absorbance of each well at 450 nm was read using a microplate reader (Molecular Devices, Sunnyvale, CA, USA) after color development (15).

The inactivated antireceptor antibodies (PAbs against BoIFNAR1 and BoIFNAR2, prepared in the laboratory previously) (11) were added to 96-well plates cultured with MDBK cells. Serial dilutions of recombinant BoIFN-χs were supplemented to the wells 2 h later. After incubation for another 24 h, 100 median tissue culture infectious doses (TCID_50_) of the VSV were supplemented to wells. Finally, the CPE was determined (9).

### Quantitative Real-Time Polymerase Chain Reaction (qPCR)

MDBK, BT, or BL cells were seeded in 12-well plates and treated with various stimulants or pretreated with 10 nM oclacitinib maleate/fludarabine (JAK1 inhibitor/STAT1 inhibitor; catalog number HY-13577A/HY-B0069; MedChem Express, Monmouth Junction, NJ, USA) for 2 h following treatment with recombinant proteins, and then, total RNAs were isolated using a RNAprep™ Pure Cell/Bacteria kit (Tiangen, Beijing, China) and reverse-transcribed into cDNAs with a PrimeScript™ RT Reagent kit with gDNA Eraser (TaKaRa Biotechnology, Dalian, China) according to the manufacturer’s instructions. Expression of related genes (**Table S1**) was determined by qPCR with a SYBR^®^ Premix ExTaq™ II (Tli RNaseH Plus) kit (TaKaRa Biotechnology) using a Real-Time PCR Detection System (7500; Thermo Scientific, Waltham, MA, USA). The following thermal cycling conditions were used: initial activation at 95°C for 30 s, 40 cycles of denaturation at 95°C for 5 s, annealing and extension at 60°C for 34 s, and a dissociation curve analysis step. Threshold cycle numbers were normalized to triplicate samples amplified with the specific primers of glyceraldehyde 3-phosphate dehydrogenase (GAPDH). The results were ananlyzed with the 2^−ΔΔCT^ or 2^−ΔCT^ method.

### Western Blotting

MDBK, EBK, BT, or BL cells were seeded in six-well plates and incubated separately with recombinant proteins for different times or pretreated with JAK1/STAT1 inhibitor following treatment with recombinant proteins for another 12 h. Then, cells were lysed with M-PER^®^ Mammalian Protein Extraction Reagent (Thermo Scientific) and mixed with 5× sodium dodecyl sulfate (SDS) loading buffer. Next, the cell mixture was resolved by 12% sodium dodecyl sulfate-polyacrylamide gel electrophoresis (SDS-PAGE) and transferred onto membranes of polyvinylidene fluoride (PVDF). Then, the PVDF membranes were blocked with 5% skimmed milk/PBST overnight at 4°C. The primary antibodies employed were antimyxovirus resistance protein 1 (Mx1) (GTX110256; GeneTex, Irvine, CA, USA) and anti-GAPDH (GTX100118; GeneTex). HRP-conjugated goat antirabbit IgG was used as the second antibody. PVDF membranes were colored with ECL substrate (Beyotime, Beijing, China).

### Dual-Luciferase Reporter Assay

BL cells were cultured in 12-well plates and transfected with 1.6 μg luciferase reporter plasmids and 0.04 μg of pRL-SV40-Renilla using Lipofectamine 3000 (Invitrogen, Carlsbad, CA, USA). After 24 h of transfection, cells were treated with various stimulants or pretreated with JAK1/STAT1 inhibitor for 2 h following treatment with recombinant proteins for another 12 h. Cell samples were lysed using a Dual-Glo™ Luciferase Assay kit (Promega, Fitchburg, WI, USA). Luciferase activities were detected by a Dual-Luciferase Reporter Assay System (GloMax 20/20) according to manufacturer (Promega) protocols.

### Statistical Analyses

Statistical analyses were carried out with Prism 6x (GraphPad, San Diego, CA, USA) using one-way analysis of variance. Data are the mean ± SD. Statistical significance was determined by the Student’s *t*-test, with *P*<0.05 indicating significance.

## Results

### Analyses of Type-I IFN Gene Loci and Location of BoIFN-χs

**Figure 1:**
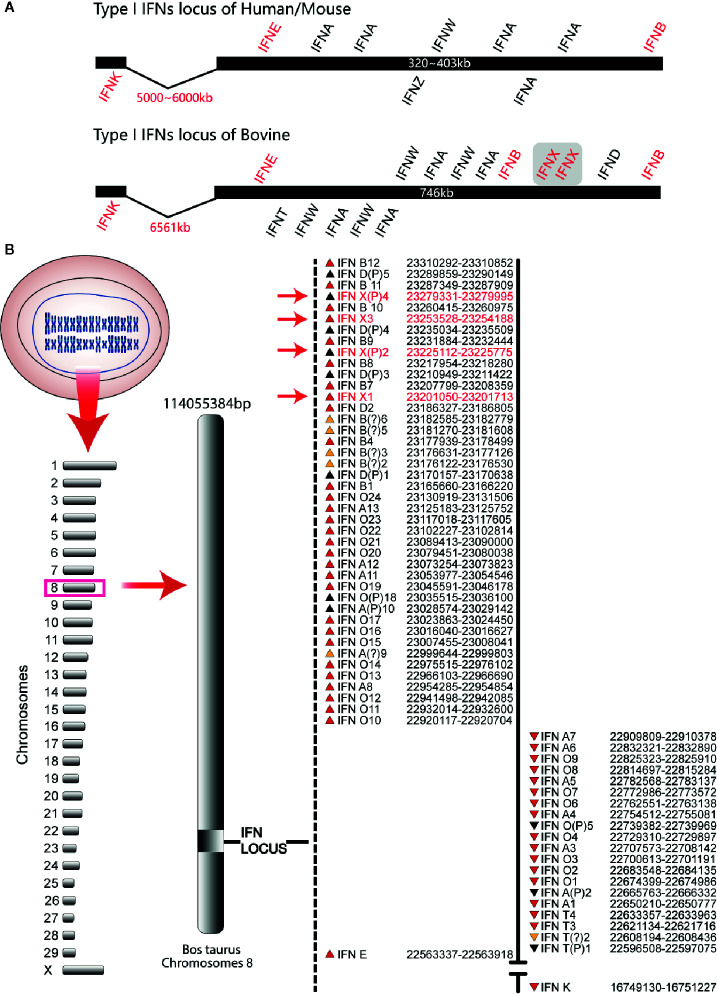
Type-I IFN locus in bovines (schematic). **(A)** Comparison of the type-I IFN locus. These schematics illustrate the conserved locus of the mouse, human, and bovine but are not drawn to scale. Each IFN gene is represented by its abbreviated name and the position above and below the schematic line represents the direction of transcription. Human/mouse type-I IFN locus was used as control. **(B)** Gene map of the bovine type-I IFN locus. The known bovine type-I IFN genes reside within two sub-loci. The position of each gene relative to the line and direction of transcription (the arrow direction) is denoted on the map. The subfamily for each gene is designated by the abbreviated name. Pseudogenes are indicated with the letter “P” after the subfamily designation. IFN-χ genes are marked with red font.

The organization of the bovine type-I IFN gene locus was similar to that of mice and humans. IFN-β (IFNB) and IFN-ϵ genes (IFNE) were distributed at the outer terminus of the locus. All the other genes were distributed between these two genes except that the IFN-κ gene (IFNK) was distributed 5000–7000 kb from IFNE. All of these genes in bovines were localized on two strands and transcribed in two opposite directions, an arrangement that is different from that in humans and mice (**Figure 1**). The bovine type-I IFN gene locus had two sub-loci encompassing 701 kb, which is about two times that of humans (403 kb) and mice (323 kb) (**Figure 1A**). There were five IFN-δ pseudogenes and four IFN-χ genes (IFNXs) in the 12 IFNBs at the end of the bovine type-I IFN gene locus. These genes had the same transcription direction, whereas the other regions of the locus were occupied by 13 IFN-αs and 24 IFN-ωs (**Figure 1B**). Based on the genetic map assigned during the assembly process and reported on the National Center for Biotechnology, the clustering of the locus of IFN genes was on bovine chromosome 8. Four IFNXs were found in the bovine genome and IFNX2 and IFNX4 were present as the pseudogenes (**Figure 1B**), which were amplified from bovine genome and sequenced, containing an early termination codon at codons 121 (IFNX2) and 17 (IFNX4).

### Molecular Characterization of BoIFN-χs

BoIFN-χ1 gene fragments and BoIFN-χ3 gene fragments cloned in our study contained a 5′-untranslated region (UTR), 3′-UTR, and an ORF (**Figure S1**). The sequences have been deposited in the NCBI GenBank database under the accession numbers KU695660 and KU695659. There are a presumptive TATA element and six IFN-induced gene regulatory motifs (GAAANN) in the 5′-UTR of BoIFN-χs (**Figure S1**), both of which are the characteristics of IFN-induced gene promoters. Bovine IFNX1 and IFNX3 were conserved with each other. They shared 95.54% nucleotide identity and 92.47% amino-acid similarity (**Figure S2A**), and exhibited 33%–55% similarity to other type-I IFNs in bovines and 68% similarity to IFNX in pigs at the amino acid level (**Figure S2B**). Bovine IFNX1 (186 amino acids) had one extra amino acid than bovine IFNX1. Bovine IFNXs contained five conserved cysteines and one signal peptide. A molecular mass of 21.129 kDa and 20.956 kDa and a theoretical isoelectric point of 7.60 and 7.17 were estimated for bovine IFNX1 and IFNX3, respectively.

A phylogenetic tree showed that bovine IFNXs genes were similar to pig IFNAW (IFNX) and horse and cat IFNA2-like predicted genes (IFNX), but not to other BoIFNs. These data suggest that bovine IFNXs evolved from a novel IFNX branch but not from the other types of IFNs (**Figure S2D**). Bovine IFNXs contained the putative IFNAR-1 binding site as well as the N- and O-linked glycosylation sites. Prediction of three-dimensional structure revealed that Bovine IFNXs contain five putative alpha helices, which is consistent with the IFN protein structure analyzed by crystal diffraction (**Figure S2C**). The molecular characteristics analyzed above are consistent with the typical characteristics of type-I IFNs.

### Protein Expression and PAbs Preparation of BoIFN-χs

SDS-PAGE results show that the recombinant proteins rHis-BoIFN-χ1, rBoIFN-χ3, and rHis-BoIFN-χ3 were expressed mainly as inclusion bodies, which revealed a molecular weight of 35, 18 and 35 kDa, respectively (**Figure S3A–C**). A single target band of rHis-BoIFN-χ1 and rHis-BoIFN-χ3 was present after purification (**Figure S3D**). The titers of PAbs against BoIFN-χ was 1:102 400 (**Figure S3E**).

### Transcription Analyses of BoIFN-χs

**Figure 2:**
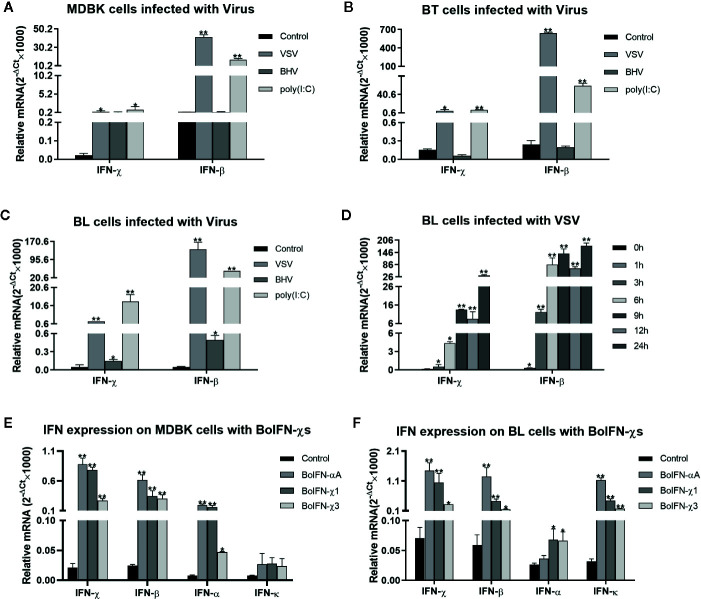
Transcription analyses of BoIFN-χs. **(A–C)** Transcription analyses of BoIFN-χs induced by viruses in MDBK/BL/BT cells. VSV (0.1 MOI), BHV (0.1 MOI), and poly(I:C) (1 μg/mL) were separately infected or transfected the cells for 6 h; the cells without treatment were used as control, and then, the RNAs were collected for qPCR. **(D)** Time dependence of BoIFN-χs transcription induced by the VSV in BL cells. VSV (0.01 MOI) were separately infected BL cells at different times, the cells without VSV infection were used as control, and then, the RNAs were collected for qPCR. **(E, F)** Transcription analyses of type-I IFNs regulated by BoIFN-χs in MDBK/BL cells. BoIFN-χ1 (5 μg/mL) and BoIFN-χ3 (5 μg/mL) were incubated in cells for 6 h, and BoIFN-αA (5 μg/mL) was used as positive control, and the cells without IFN incubation were used as mock-treated control. Data are the mean ± SD (n = 3) of one representative experiment. *P < 0.05, **P < 0.01.

The transcription of BoIFN-χs could be upregulated by the VSV, BHV, and poly(I:C) in MDBK, BT, and BL cells. This upregulation was significant with induction with the VSV and poly(I:C), and the regulation induced by the VSV on BL cells was dependent upon time (**Figures 2A–D**). BoIFN-χs could also induce transcription of IFN-β, IFN-α, IFN-χ, and IFN-κ in MDBK and BL cells (**Figures 2E, F**).

### Biologic Characteristics of BoIFN-χ

**Figure 3:**
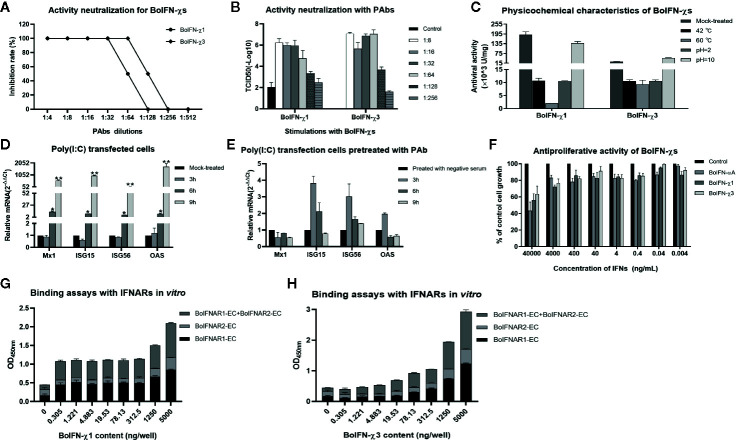
Biologic characteristics of BoIFN-χs. Neutralization of antiviral activity with PAbs against BoIFN-χs with CPE **(A)** and TCID50 measurement **(B)**. Serial dilution of inactive PAbs against BoIFN-χs were incubated with BoIFN-χs at 37°C for 2 h, and then, the mixtures were centrifuged for collection of supernatant and deterimination of the CPE, or the above cells were collected for further measurement of the TCID50. **(C)** Sensitivity to pH and temperature of BoIFN-χs. **(D)** qPCR of ISGs in BL cells with poly(I:C) transfection. **(E)** qPCR of ISGs in BL cells with poly(I:C) transfection after PAbs against BoIFNχs pretreatment. BL cells were pretreated with inactive PAbs against BoIFNχs for 2 h, and poly(I:C) (1 μg/mL) was transfected in the cells for another 3, 6, or 9 h, and then, the cells were collected for qPCR. The preimmune serum was used as control. **(F)** Antiproliferative activity of BoIFN-χs in MDBK cells. Relative proliferation was determined as a percentage of the control (0 μg/mL). **(G)** ELISA for the binding ability of BoIFN-χ1 to BoIFNAR. **(H)** ELISA for the binding of BoIFN-χ3 to BoIFNAR. Data are the mean ± SD (n = 3) of one representative experiment. *P < 0.05, **P < 0.01.

BoIFN-χ1 and BoIFN-χ3 exerted antiviral activities against the VSV in MDBK, EBK, BT, BL, and PK-15 cells, but not in BHK21 and MDCK cells or against the BHV in BL cells. BoIFN-χ1 exerted higher antiviral activity than that of BoIFN-χ3, whereas their antiviral activities were lower than those of BoIFN-αA (**Table 1**). The antiviral activities of BoIFN-χ1 and BoIFN-χ3 could be abrogated completely by PAbs against BoIFN-χs at a dilution of 1:32 and 1:64, respectively (**Figures 3A, B**).

**Table 1:** Antiviral activity of BoIFN-χs.

Measurement System	Antiviral activity (U/mg)		
	IFN-αA	IFN-χ1	IFN-χ3
MDBK-VSV	2.38×10^5^	2.03×10^5^	2.53×10^4^
EBK-VSV	2.90×10^6^	1.01×10^5^	1.13×10^4^
BT-VSV	8.25×10^6^	4.03×10^5^	1.29×10^5^
BL-VSV	6.24×10^5^	2.46×10^3^	1.20×10^4^
PK 15-VSV	3.90×10^5^	2.02×10^3^	1.75×10^3^
BHK 21-VSV	0	0	0
MDCK-VSV	0	0	0
BL-BEV	6.74×10^6^	1.28×10^5^	7.12×10^4^

Antiviral activity was completely absent after treatment with 0.25% trypsin (data not shown). Antiviral activity remained after treatment at pH 2.0, 10.0, or with increasing temperature (**Figure 3C**). Poly(I:C) could significantly induce ISG transcription after transfection for 6 and 12 h (**Figure 3D**) while the Mx1 and OAS transcription induced by poly(I:C) was downregulated at 6 and 12 h after the PAbs against BoIFN-χs pretreatment compared with preimmune serum pretreatment (**Figure 3E**). BoIFN-χ1 and BoIFN-χ3 revealed dose-dependent antiproliferative activities in MDBK cells. This result suggests that there was no cytotoxicity at the concentrations tested (even at a concentration of 8.12×10^3^ U/mL for BoIFN-χ1 and 1.01×10^3^ U/mL for BoIFN-χ3) in MDBK cells (**Figure 3F**).

### BoIFN-χs Signal Through the JAK-STAT Pathway

#### BoIFN-χ Binds With Type-I IFN Receptors

BoIFN-χs could combine with BoIFNAR1 and BoIFNAR2 in a dose-dependent manner, and BoIFNAR1 had a stronger combination with BoIFN-χs than that of BoIFNAR2 (**Figures 3G, H**). PAbs against BoIFNAR could block the antiviral activities of BoIFN-χs on MDBK cells in the dose-dependent manner, and the blocking effect of the antibody against BoIFNAR2 was much stronger (data not shown).

#### BoIFN-χ-Induced Expression of ISGs

**Figure 4:**
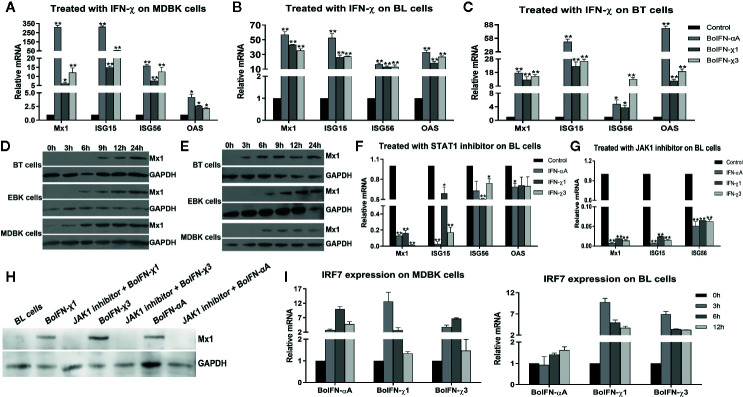
ISG expression induced with BoIFN-χs. **(A–C)** Transcription of ISGs in MDBK/BL/BT cells with BoIFN-χs treatment. BoIFN-χ1 (5 μg/mL) and BoIFN-χ3 (5 μg/mL) were separately incubated in cells for 6 h, and then, the cells were collected for qPCR. BoIFN-αA (5 μg/mL) was used as positive control, and the cells without IFN incubation were used as a mock-treated control. **(D, E)** Western blotting of expression of Mx1 protein upon BoIFN-χs treatment. BoIFN-χ1 (5 μg/mL) and BoIFN-χ3 (5 μg/mL) were separately incubated in cells for 0–24 h, and then the cells were collected for western blotting. **(F, G)** Transcription of ISGs in BL cells upon BoIFN-χs treatment followed by treatment with a STAT1/JAK1 inhibitor. BL cells were pretreated with STAT1/JAK1 inhibitor for 2 h, and then, the BoIFN-χ1 (5 μg/mL) and BoIFN-χ3 (5 μg/mL) were separately added in cells for another 6 h, the cells were collected for qPCR. BoIFN-αA (5 μg/mL) was used as positive control, and the cells without inhibitors were used as mock-treated control. **(H)** Western blotting of expression of Mx1 protein upon BoIFN-χs treatment followed by treatment with a JAK1 inhibitor. BL cells were pretreated with JAK1 inhibitor for 2 h, and then, the BoIFN-χ1 (5 μg/mL) and BoIFN-χ3 (5 μg/mL) were separately added in cells for another 12 h, and the cells were collected for western blotting. BoIFN-αA (5 μg/mL) was used as positive control. **(I)** Transcription of IRF7 in MDBK/BL cells upon BoIFN-χs treatment for 3–12 h. GAPDH was used as the internal reference control, and cells without IFN treatment were used as the mock-treated control. Data are the mean ± SD (n = 3) of one representative experiment. *P < 0.05, **P < 0.01.

BoIFN-χs could induce transcription of Mx1, ISG15, ISG56, and 2’-5’-oligoadenylate synthetase (OAS) in MDBK, BL, and BT cells to different extents (**Figures 4A–C**). BoIFN-χs could induce transcription of IRF7 in a time-dependent manner in MDBK and BL cells (**Figure 4I**).

BoIFN-χs could induce expression of Mx1 on BT, EBK, and MDBK cells in a time-dependent manner. Such induction of expression started from 3 h in MDBK cells, 6 h in EBK cells, and 9 h in BT cells with BoIFN-χ1 treatment. The start of Mx1 expression in BT cells treated with BoIFN-χ1 was 6 h later than that of BoIFN-χ3 (**Figures 4D, E**).

#### BoIFN-χ Signals Through JAK1 and STAT1

**Figure 5:**
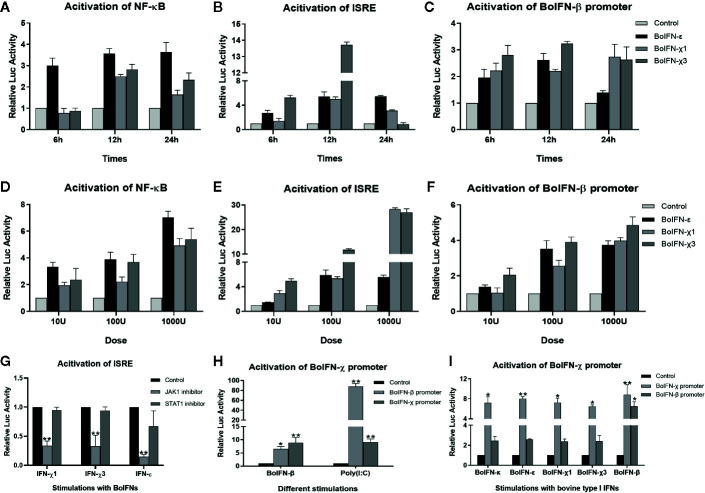
Promoter activation with BoIFN-χs. **(A–F)** Time/dose dependence of activation of promoters of BoIFN-β (pGL3-BoIFNβ)/ISRE (pISRE-TA-Luc)/NF-κB (pNFκB-TA-Luc) upon BoIFN-χs treatment. BL cells were transfected with 1.6 μg pGL3-BoIFNβ/pISRE-TA-Luc/pNFκB-TA-Luc and 0.04 μg of pRL-SV40-Renilla for 24 h, and then, cells were incubated with a different dose of BoIFN-χ1 and BoIFN-χ3 for different times. Then, the cells were collected for dual-luciferase reporter assay. BoIFN-ϵ was used as positive control. **(G)** Activation of the ISRE (pISRE-TA-Luc) promoter in BL cells upon BoIFN-χs treatment followed by treatment with a STAT1/JAK1 inhibitor. BL cells were pretreated with JAK1/STAT1 inhibitor for 2 h following treatment with BoIFN-χ1 (5 μg/mL) and BoIFN-χ3 (5 μg/mL) for another 12 h, and the cells were collected for dual-luciferase reporter assay. BoIFN-ϵ (5 μg/mL) was used as a positive control, and the cells without inhibitors were used as a mock-treated control. **(H)** Activation of the BoIFN-χ (pGL3-BoIFNχ) promoter. BL cells were transfected with 1.6 μg pGL3-BoIFNχ/pGL3-BoIFNβ and 0.04 μg of pRL-SV40-Renilla for 24 h, and then. BoIFN-β (5 μg/mL)/poly(I:C) (1 μg/mL) were incubated for another 12 h, and the cells were collected for dual-luciferase reporter assay. The cells transfected with pGL3-BoIFNχ were used as positive control, and the cells transfected with pGL3-basic vector were used as mock-treated control. **(I)** Activation of the BoIFN-χ promoter with bovine type-I IFNs. The wells without IFN treatment were used as the mock-treated control. Error bars indicate the standard deviation of three replicates. *P < 0.05, **P < 0.01.

After pretreatment with the JAK1/STAT1 inhibitor oclacitinib maleate/fludarabine, expression of ISGs upon BoIFN-χs treatment could be downregulated significantly in BL cells (**Figure 4F–H**), and activation of ISRE could be reduced compared with IFN control without inhibitor (**Figure 5G**).

#### BoIFN-χ Induced Activation of the Promoters of NF-κB, ISRE, and BoIFN-β

In comparison with those in mock-treated cells, the promoters of NF-κB, ISRE, and BoIFN-β could be activated upon BoIFN-χs treatment in dose- and time-dependent manners. Such activation peaked 12 h after stimulation with IFNs at 1000 U. There was no significant difference between BoIFN-χ1 and BoIFN-χ3 for activation of the promoters of NF-κB, ISRE, and BoIFN-β (**Figures 5A–F**).

#### BoIFN-χ Promoters Were Activated Upon Treatment With Type-I IFNs

BoIFN-χ promoters could be activated upon treatment with BoIFN-β or poly(I:C) (**Figure 5H**). The BoIFN-χ promoter could be activated upon treatment with bovine type-I IFNs (**Figure 5I**).

## Discussion

IFNs are a family of cytokines that are essential for the antiviral response in vertebrates. The type-I IFN family includes α, β, ω, κ, ϵ, and τ subclasses, which are clustered according to sequence similarities and binding to the same cell surface receptor (3). Throughout the bovine type-I IFN gene locus, the number of genes is significantly more than that of humans and mice, and new subtypes with distinct characteristics have arisen during evolution.

IFN-χ has been characterized in pigs; it has only one single copy and exerts activity against the FMDV (8). IFN-χ exists as a subfamily in bovines, which consists of two functional genes (BoIFN-χ1 and BoIFN-χ3) and two pseudogenes. The origin of the IFN-χ gene is not known yet, but it appears to constitute a unique IFN subfamily whose closest relatives are IFN-α and IFN-ω according to sequence alignments. With respect to amino acid similarity in bovines, IFN-χ shares >54% with IFN-α, ~51% with IFN-ω, and ~24% with IFN-β. This obvious difference makes IFN-χ a new subtype of the IFN classification system. In addition, BoIFN-χs have the typical molecular characteristics as those of type-I IFNs.

Comprehensive analyses of the GenBank database reveals that the IFN-χ gene could be located in 35 species, including those in the orders Insectivora, Chiroptera, Carnivora, Artiodactyla, Perissodactyla, Hyracoidea, Proboscidea, and Scandentia, all of which belong to the highly evolved Eutheria clades of the class Mammalia. The IFN-χ gene could not be found in the original subclasses Prototheria and Metatheria, suggesting that IFN-χ may originate from the early stage of Eutheria of Mammalia. IFN-χ is a new subtype of type-I IFNs produced at an important branch point during the evolution of mammals, suggesting that IFN-χ may have an important function in species evolution. BoIFN-χ has evolved into a multigene family (2), which can be divided into one separate branch of type-I IFNs in phylogenetic analyses. This branch is located in the same branch as the IFN-χs of other species: horses (6), dogs, and pigs (8).

BoIFN-χ is a key intermediate in antiviral response, which could be induced with VSV infection at the early and late phases and could induce the transcription of other type I IFNs. PAbs against BoIFN-χs could downregulate the transcriptional activation of ISGs induced by poly(I:C). BoIFN-χ has similar biologic activities with other type-I IFNs, including antiviral activities and regulation of the ISGs related to antiviral activity, which could protect against VSV infection in MDBK, EBK, BT, BL, and PK-15 cells as well as protect against BEV infection in BL cells, whereas no effects were observed in MDCK or BHK-21 cells. The antiviral activity measured with the MDBK/VSV system could be neutralized by PAbs against BoIFN-χs, suggesting this antiviral activity is specific. In addition, BoIFN-χs had antiproliferative activity and no cytotoxicity even at a high concentration in MDBK cells.

IFNs provide the first line of defense against viral infection. Their binding with the corresponding receptors initiates a signaling cascade that leads to the induction of a cellular antiviral response. IFN-α and IFN-β exhibit antiviral activity by binding with the IFNAR and then signal through the JAK-STAT pathway to stimulate expression of hundreds of downstream ISGs (16, 17). ISGs can interfere with different stages of viral life cycles to block viral transcription, degrade viral RNA, inhibit translation, and modify protein function to resist and control pathogens (18, 19). In the present study, the antiviral activity of BoIFN-χs could be blocked by PAbs against BoIFNAR, suggesting that BoIFN-χs interact with type-I IFN receptors to initiate antiviral signal transduction. BoIFN-χs could induce expression of IRF7, ISGs, and type-I IFNs, which could produce more type-I IFNs and ISGs to inhibit viral replication and exert antiviral activity. After inhibition of JAK1 or STAT1 expression with inhibitors, ISG expression was downregulated significantly, suggesting that BoIFN-χs exerts antiviral activity depending on JAK1 and STAT1 activation.

NF-κB has a key role in the early phase of production of type-I IFNs and subsequent ISGs production, thereby limiting virus replication (20), IFN-β has an essential role in the positive feedback that produces more quantity IFNs (21). IFN activation of genes bearing an ISRE is regulated through binding of ISG factors to the ISRE found in many ISGs (22). In the present study, BoIFN-χs could activate the promoters of NF-κB, ISRE, and BoIFN-β in dose- and time-dependent manners, suggesting that BoIFN-χs may play an important part in the induction of IFN-β production to produce more type-I IFNs. JAK1 or STAT1 inhibitors could inhibit the activation of ISRE, leading to the downregulation of ISGs expression. In addition, the BoIFN-χ promoter could be activated by type-I IFNs, and the amount of IFNs were induced with BoIFN-χs, suggesting that BoIFN-χs could be produced in a positive feedback of IFN production.

**Figure 6:**
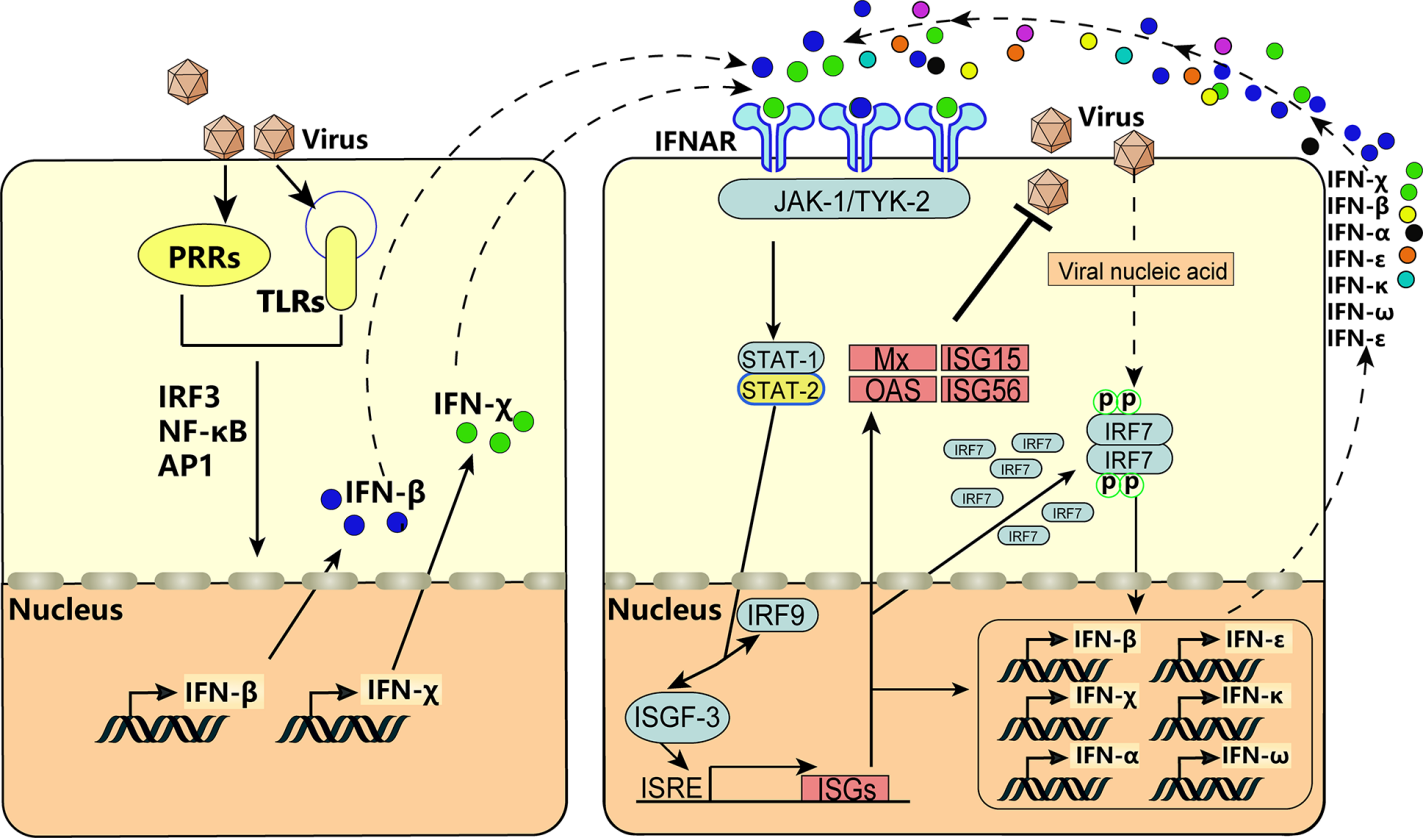
IFN-χ involvement in the positive self-feedback regulation of IFN production. Virus infection is sensed by PRRs and TLRs, and the key adaptor molecules (e.g., IRF3, NF-κB, AP1) are activated and formed into complexes. Then, the complexes translocate to the nucleus to act on the IFN-β and IFN-χ. This action results in the production of IFN-β and IFN-χ, which act on neighboring cells by binding to IFNAR and activate receptor-associated TYK2 and JAK1. This is followed by tyrosine phosphorylation of STAT1 and STAT2, which leads to formation of the heterotrimeric ISGF3 transcription factor, together with IRF-9. These transcriptional–activator complexes translocate to the nucleus and activate the ISRE, leading to induction of ISGs, activation of IRF7, and production of IFNs, thereby forming positive-feedback regulation.

After infection with VSV, BEV, or transfection with poly(I:C), expression of BoIFN-χ and BoIFN-β was upregulated significantly (**Figures 2A–D**). Then, BoIFN-χs was bound with IFNAR (**Figures 3E, F**) and activated receptor-associated TYK2 and JAK1 (**Figures 4F, G**) and formed transcriptional–activator complexes (**Figure 6**). The latter translocated to the nucleus and activated the promoters of NF-κB, ISRE, BoIFN-β, and BoIFN-χ (**Figure 5**), which induced further expression of ISGs (**Figures 4A–E**), type-I IFNs (**Figures 2E, F**) and IRF7 (**Figure 4I**) to initiate positive-feedback regulation to produce more IFNs (**Figure 6**).

## Conclusions

We analyzed the location, characteristics, and signaling pathways of BoIFN-χs. BoIFN-χs exist as a multigene family in the locus of type-I IFNs. BoIFN-χs present the typical molecular characteristics and biologic activity of other type-I IFNs. BoIFN-χs signal through the JAK-STAT pathway to establish an “antiviral state” within host cells, which is also involved in positive feedback to produce more IFNs.

## Data Availability Statement

The original contributions presented in the study are included in the article/**Supplementary Material**, further inquiries can be directed to the corresponding author/s.

## Ethics Statement

The animal study was reviewed and approved by Laboratory Animal Ethical Committee of Northeast Agricultural University.

## Author Contributions

YG designed the experiments, performed most of the experiments, analyzed the results, drafted and revised the manuscript. CL and YY performed the western blotting, plasmids construction, as well as the complementation of the revised manuscript. ZS and HD performed part of antiviral activity analysis and signal pathway analysis. XL and YW performed part of the molecular clone, protein expression, antibody preparation, and dual-luciferase reporter assay. JW and MG. designed the experiments and supervised the work. All authors contributed to the article and approved the submitted version.

## Funding

This study was supported by China Agriculture Research System (CARS-36) and National Natural Science Foundation of China (32002296).

## Conflict of Interest

The authors declare that the research was conducted in the absence of any commercial or financial relationships that could be construed as a potential conflict of interest.

## Supplementary Material

Supplementary Figure 1 Sequence analyses of bovine IFNXs. Sequence analyses of bovine IFNX1 **(A)** and IFNX3 **(B)**. The ORF regions of bovine IFNXs are depicted in uppercase. The conserved cysteines are indicated by shaded circles. Putative TATA are boxes. The potential IFN regulatory factor-binding regions conforming to GAAANN consensus sequences are underlined.

Supplementary Figure 2 Molecular characteristics of bovine IFNXs. **(A)** Alignment between bovine IFNX1 and IFNX3. Signal cleavage occurs between Gly-23 and Cys-24 (arrow). **(B)**) Amino-acid identity between the bovine type-I IFN family based on mature protein sequences. **(C)** Predicted three-dimensional structures of bovine IFNXs. **(D)** Phylogenetic analyses of bovine IFNXs. A phylogenetic tree was constructed with MEGA 7.0 using the method of UPGMA. The percentage of replicate trees in which the associated taxa are clustered together in the bootstrap test (500 replicates) are shown next to the branches. The tree is drawn to scale with branch lengths in the same units as those of the evolutionary distances used to infer the phylogenetic tree.

Supplementary Figure 3 SDS-PAGE of recombinant BoIFN-χs protein. **(A)** SDS-PAGE of rHis-BoIFN-χ1. Lane M: unstained protein marker; Lane 1: negative control of pET32a (+) induction; Lane 2: rHis-BoIFN-χ1 after induction; Lane 3: supernatants of rHis-BoIFN-χ1; Lane 4: sedimentations of rHis-BoIFN-χ1. **(B)** SDS-PAGE of rBoIFN-χ3. Lane M: unstained protein marker; Lane 1: sedimentations of rBoIFN-χ3; Lane 2: supernatants of rBoIFN-χ3; Lane 3: rBoIFN-χ3 after induction; Lane 4: negative control of pET30a (+) induction. **(C)** SDS-PAGE of rHis-BoIFN-χ3. Lane M: unstained protein marker; Lane 5: negative control of pET32a (+) induction; Lane 6: rHis-BoIFN-χ3 after induction; Lane 7: supernatants of rHis-BoIFN-χ3; Lane 8: sedimentations of rHis-BoIFN-χ3. **(D)** SDS-PAGE of purified rHis-BoIFN-χs. Lane M: unstained protein marker; Lane 1: purified rHis-BoIFN-χ1; Lane 2: purified rHis-BoIFN-χ3. **(E)** The titers of PAbs against BoIFNχs.
