# Supplementary material for: A Novel Type-I Interferon Family, Bovine Interferon-Chi, Is Involved in Positive-Feedback Regulation of Interferon Production
Source: Front Immunol. 2020 Oct 30;11:528854. doi: 10.3389/fimmu.2020.528854 (PMC7662560; doi:10.3389/fimmu.2020.528854)
Supplement: Supplementary file 1 [file Table_1.docx]

Table S1. Nucleotide Sequence of the PCR primers used in this study

| Primer | Sequence (5’-3’) | Objective |
| --- | --- | --- |
| BoIFNχ1-F | GGCGACTTGTGGATTCTT | BoIFNχ1 gene clone |
| BoIFNχ1-R | TCACATGGCAGCTACTGGT |  |
| BoIFNχ3-F | CCTCAGTATCCCTGAGTGTATGG | BoIFNχ3 gene clone |
| BoIFNχ3-R | GTCACATGGCAGCTACCAGG |  |
| BoNX1-BIF | TCCGGATCC*_Bam_*_HI_TGTGAGCTGCCTGCGAGC | Protein plasmid construction of BoIFNχ1 |
| BoNX1-XIR | ACACTCGAG*_Xho_*_I_TTATTTCCTGAGTTTTCCGATG |  |
| BoNX3-BIF | TCCGGATCC*_Bam_*_HI_TGTGAGCTGCCTTCGAGC | Protein plasmid construction of BoIFNχ1 with the common reverse primer |
| BoNX3-NIF | TCCCATATG*_Nde_*_I_TGTGAGCTGCCTTCGAGC |  |
| BoNX3-XIR | ACACTCGAG*_Xho_*_I_TTATTTCCTGAGTTTTCCAAT |  |
| BoIFNχ-qF | CTTCCAGCTCTTCAGCACCACG | Real time PCR for BoIFN-χ |
| BoIFNχ-qR | TGACAACCTCCCAGGCACAG |  |
| BoIFNβ-qF | CAGCACATCTTCGGCATTCTC | Real time PCR for BoIFN-β |
| BoIFNβ-qR | GACGATTCATCTGCCCATAG |  |
| BoIFNα-qF | GTGAGGAAATACTTCCACAGACTCACT | Real time PCR for BoIFN-α |
| BoIFNα-qR | TGARGAAGAGAAGGCTCTCATGA |  |
| BoIFNκ-qF | GAAAGTATGTGTGGCTGGCGTG | Real time PCR for BoIFN-κ |
| BoIFNκ-qR | GCAGAGGCTGGGTGTATGAG |  |
| BoIRF7-qF | CCGCACTACACCATCTACTTG | Real time PCR for IRF7 |
| BoIRF7-qR | CCAGGACCAGGCTCTTCTC |  |
| BoMx1-qF | TCAACCTCCACCGAACTG | Real time PCR for Mx1 |
| BoMx1-qR | TCTTCTTCTGCCTCCTTCTC |  |
| BoISG15-qF | GCAGCCAACCAGTGTCTG | Real time PCR for ISG15 |
| BoISG15-qR | CCTAGCATCTTCACCGTCAG |  |
| BoISG56-qF | TGGACTGTGAGGAAGGATGG | Real time PCR for ISG56 |
| BoISG56-qR | AGGCGATAGACAACGATTGC |  |
| BoOAS-qF | TTCGGTCATCTTGCTCTCAG | Real time PCR for OAS |
| BoOAS-qR | GTCTATCTCAACAGTCACAATCC |  |
| BoGAPDH-qF | TTCAACGGCACAGTCAAGG | Real time PCR for GAPDH |
| BoGAPDH-qR | ACATACTCAGCACCAGCATCAC |  |
| BoIFNX-PTDF | CTGATGGGAAGAGCCCACT | BoIFNχs promoter gene clone |
| BoIFNX-PTDR | TCTCTCCATCTGACTCCAACG |  |
| BoIFNX-PTF | GCAGGTACC*_Kpn_*_I_CTGAAGCAAAGTATCCTGTG | Reporter plasmid construction of BoIFNχs |
| BoIFNX-PTR | TTGCTCGAG*_Xho_*_I_TGGGGGCTCTCAGGACTGCAT |  |

Note: The restriction enzyme sites that were introduced in primers are underlined.
