# Supplementary figures and images for: A Novel Type-I Interferon Family, Bovine Interferon-Chi, Is Involved in Positive-Feedback Regulation of Interferon Production
Source: Front Immunol. 2020 Oct 30;11:528854. doi: 10.3389/fimmu.2020.528854 (PMC7662560; doi:10.3389/fimmu.2020.528854)

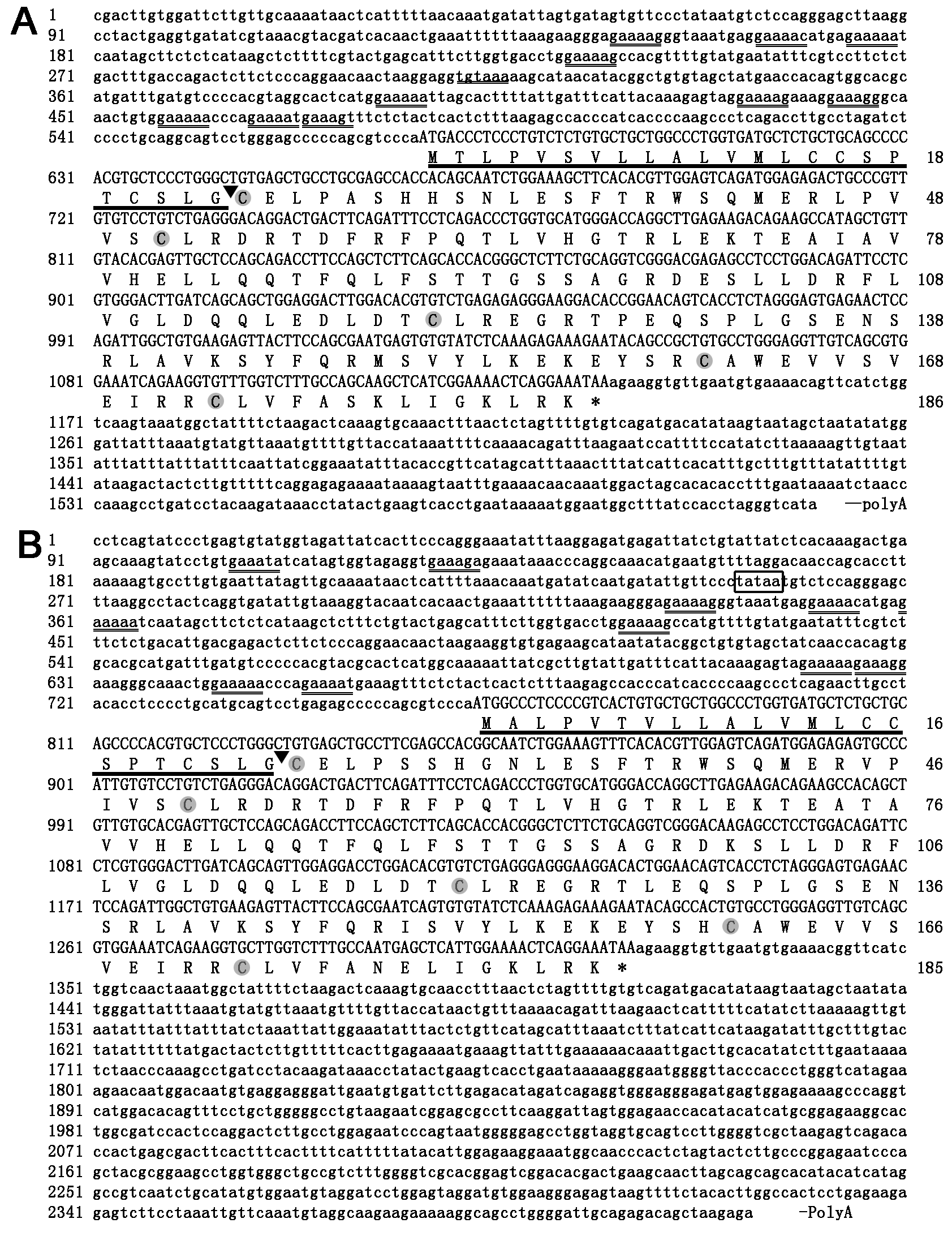

Supplement: Supplementary Figure 1 — Sequence analyses of bovine IFNXs. Sequence analyses of bovine IFNX1 (A) and IFNX3 (B). The ORF regions of bovine IFNXs are depicted in uppercase. The conserved cysteines are indicated by shaded circles. Putative TATA are boxes. The potential IFN regulatory factor-binding regions conforming to GAAANN consensus sequences are underlined. [file Image_1.tif]

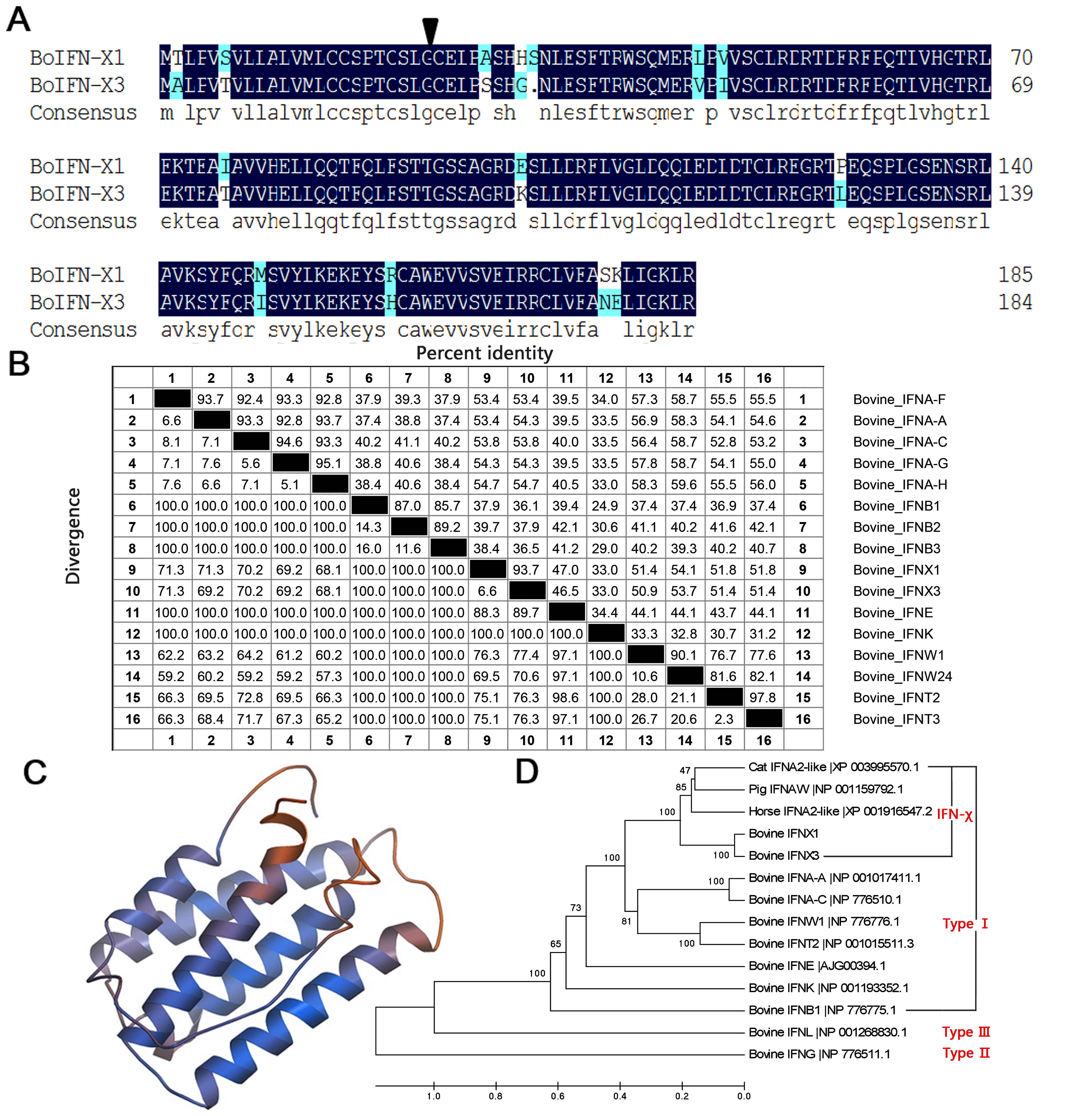

Supplement: Supplementary Figure 2 — Molecular characteristics of bovine IFNXs. (A) Alignment between bovine IFNX1 and IFNX3. Signal cleavage occurs between Gly-23 and Cys-24 (arrow). (B)) Amino-acid identity between the bovine type-I IFN family based on mature protein sequences. (C) Predicted three-dimensional structures of bovine IFNXs. (D) Phylogenetic analyses of bovine IFNXs. A phylogenetic tree was constructed with MEGA 7.0 using the method of UPGMA. The percentage of replicate trees in which the associated taxa are clustered together in the bootstrap test (500 replicates) are shown next to the branches. The tree is drawn to scale with branch lengths in the same units as those of the evolutionary distances used to infer the phylogenetic tree. [file Image_2.tif]

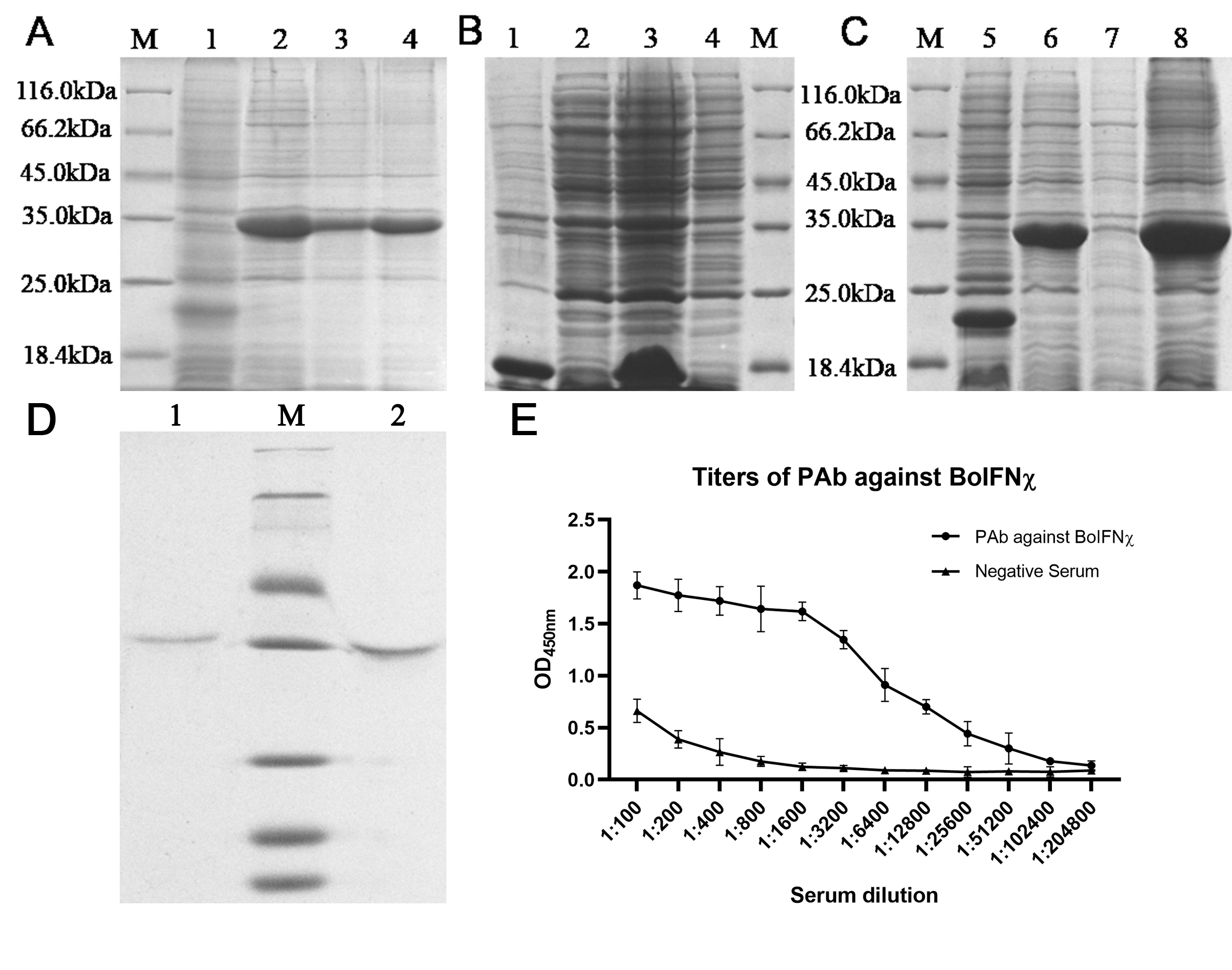

Supplement: Supplementary Figure 3 — SDS-PAGE of recombinant BoIFN-χs protein. (A) SDS-PAGE of rHis-BoIFN-χ1. Lane M: unstained protein marker; Lane 1: negative control of pET32a (+) induction; Lane 2: rHis-BoIFN-χ1 after induction; Lane 3: supernatants of rHis-BoIFN-χ1; Lane 4: sedimentations of rHis-BoIFN-χ1. (B) SDS-PAGE of rBoIFN-χ3. Lane M: unstained protein marker; Lane 1: sedimentations of rBoIFN-χ3; Lane 2: supernatants of rBoIFN-χ3; Lane 3: rBoIFN-χ3 after induction; Lane 4: negative control of pET30a (+) induction. (C) SDS-PAGE of rHis-BoIFN-χ3. Lane M: unstained protein marker; Lane 5: negative control of pET32a (+) induction; Lane 6: rHis-BoIFN-χ3 after induction; Lane 7: supernatants of rHis-BoIFN-χ3; Lane 8: sedimentations of rHis-BoIFN-χ3. (D) SDS-PAGE of purified rHis-BoIFN-χs. Lane M: unstained protein marker; Lane 1: purified rHis-BoIFN-χ1; Lane 2: purified rHis-BoIFN-χ3. (E) The titers of PAbs against BoIFNχs. [file Image_3.tif]
